# Harringtonine Inhibits Herpes Simplex Virus Type 1 Infection by Reducing Herpes Virus Entry Mediator Expression

**DOI:** 10.3389/fmicb.2021.722748

**Published:** 2021-08-31

**Authors:** Ye Liu, Qiao You, Fang Zhang, Deyan Chen, Zhenping Huang, Zhiwei Wu

**Affiliations:** ^1^Medical School of Nanjing University, Nanjing, China; ^2^Department of Ophthalmology, Jinling Hospital, School of Medicine, Nanjing University, Nanjing, China; ^3^Jiangsu Key Laboratory of Molecular Medicine, Medical School, Nanjing University, Nanjing, China; ^4^State Key Laboratory of Analytical Chemistry for Life Science, Nanjing University, Nanjing, China; ^5^School of Life Sciences, Ningxia University, Yinchuan, China

**Keywords:** harringtonine, herpes simplex virus type 1, acyclovir-resistant, herpes virus entry mediator, entry receptor

## Abstract

Herpes simplex virus type 1 (HSV-1) infection induces various clinical disorders, such as herpes simplex encephalitis (HSE), herpes simplex keratitis (HSK), and genital herpes. In clinical intervention, acyclovir (ACV) is the major therapeutic drug used to suppress HSV-1; however, ACV-resistant strains have gradually increased. In the present study, harringtonine (HT) significantly inhibited infection of HSV-1 as well as two ACV-resistant strains, including HSV-1 blue and HSV-1 153. Time-of-drug addition assay further revealed that HT mainly reduced the early stage of HSV-1 infection. We also demonstrated that HT mainly affected herpes virus entry mediator (HVEM) expression as shown by qPCR, Western Blot, and *Immunofluorescence*. Collectively, HT showed antiviral activity against HSV-1 and ACV-resistant strains by targeting HVEM and could be a promising therapeutic candidate for mitigating HSV-1-induced-pathogenesis.

## Introduction

Herpes simplex virus type 1 (HSV-1) is a double-stranded DNA virus belonging to *Herpesvirus*, spreading worldwide. The estimated prevalence of HSV-1 infection is 67% globally, ranging from 49 to 87% (Looker et al., 2015; Harris, 2019). The majority of HSV-1 infections occur during childhood by oral-oral contact (Gupta et al., 2007). After that, HSV-1 most likely establishes lifelong latency in the trigeminal ganglion (Pires de Mello et al., 2016). Furthermore, recurrent infections of HSV-1 can cause complications such as orofacial herpes, ocular lesions, genital diseases, and in severe cases, lethal encephalitis (Levitz, 1998; Kaye and Choudhary, 2006; Gupta et al., 2007). Acyclovir (ACV), introduced in 1977, is the first specific drug for HSV treatment by targeting viral DNA polymerase (Elion et al., 1977). Until now, ACV and its analogs remain the first-line treatment for the management of HSV-1 infection. However, with the increasing number of immunocompromised individuals and the prolonged administration of antiviral agents, ACV resistance emerges (Andrei and Snoeck, 2013; Piret and Boivin, 2014, 2016). Therefore, the development of novel and potent antiviral treatment with different mechanisms is imminent (Jiang et al., 2016).

A mature HSV-1 virion is a spherical particle ~186nm in diameter, composed of a core containing viral linear dsDNA, an icosahedra capsid, a tegument, and a lipid bilayer envelope (Whitley and Roizman, 2001; Heming et al., 2017; Ahmad and Wilson, 2020). HSV-1 enters the host cells mainly through the fusion of the viral envelope with the plasma membrane and transports the viral capsid to the nucleus (Atanasiu et al., 2010; Kukhanova et al., 2014; Connolly et al., 2021). Successful HSV-1 fusion requires multiple viral glycoproteins and cellular receptors to interact in a sequential process. Among these, HSV-1 glycoprotein D (gD) binding to its receptor and undergoing a conformational change is the first step (Yasushi et al., 2018). gD can bind to three types of receptors: herpes virus entry mediator (HVEM; Montgomery et al., 1996), nectin-1 (Geraghty et al., 1998) and nectin-2 (Warner et al., 1998), and 3-O-sulfated heparan sulfate (3-O-S HS; Shukla et al., 1999). HVEM and nectin-1 are the major receptors mediating HSV-1 entry into human cells (Petermann et al., 2015). HVEM is a member of the tumor necrosis factor receptor superfamily and is expressed in many tissues (Steinberg et al., 2008). In addition to influencing virus entry, HVEM also plays a role in the pathogenesis of HSV-1 infection, including innate responses, chronic inflammation, latency, and reactivation cycle (Edwards and Longnecker, 2017; Park et al., 2020; Tormanen et al., 2020). Identified HVEM ligands come from two distinct types of families, the TNF-related cytokines, LIGHT, and Lymphotoxin-a, and the Ig-related membrane proteins, B and T lymphocyte attenuator (BTLA), and CD160 (Cai and Freeman, 2009; Murphy and Murphy, 2010). Interactions with these immune factors help HVEM regulate proinflammatory and inhibitory signaling and activate NF-κB survival programs (Ware and Sedy, 2011). Thus, HVEM may play a diverse and important role in the process of HSV-1 infection.

**Figure 1:**
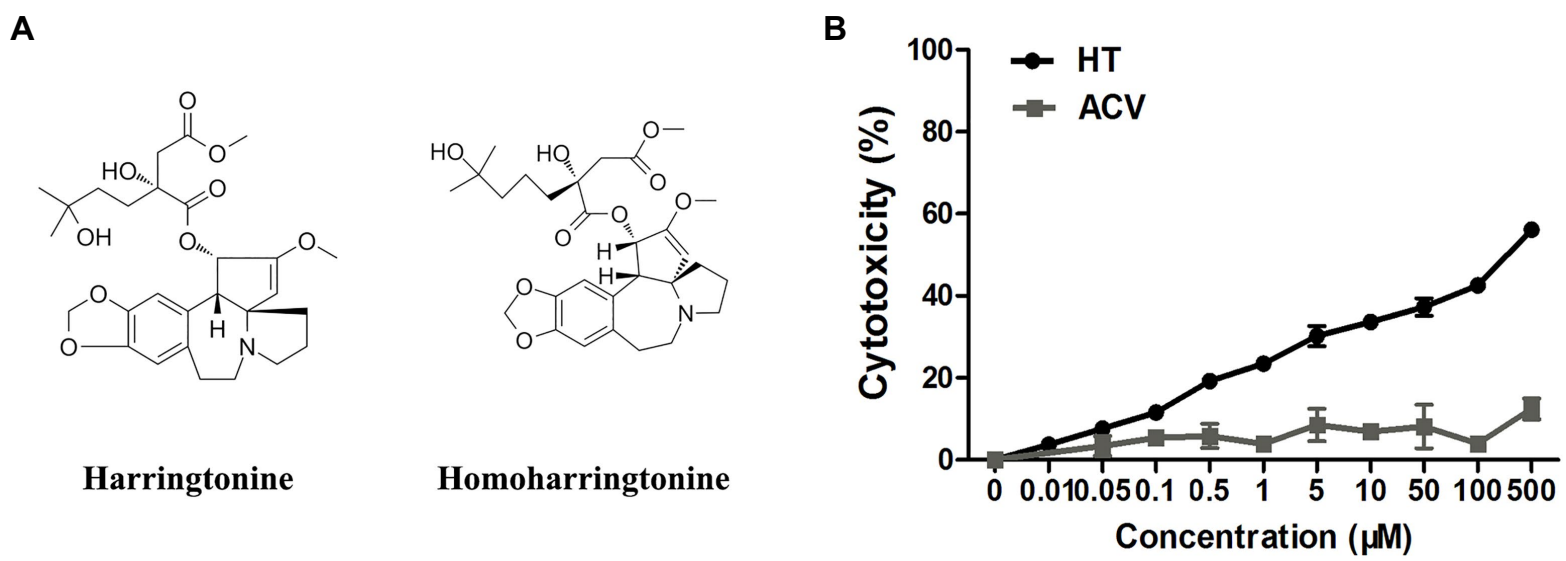
**(A)** The structures of harringtonine (HT) and homoharringtonine (HHT). **(B)** The cytotoxicity of HT and acyclovir (ACV) on Vero cells for 48h was determined by Cell Counting Kit 8 (CCK-8) assays.

Harringtonine (HT), a natural alkaloid, was first isolated from Cephalotaxus harringtonia in 1963, alone with homoharringtonine (HHT), isoharringtonine, and cephalotaxine (CET). HT and HHT are homologs, except that HHT has one more methylene group in the side chain (Figure 1A; Powell et al., 1970; Lu and Wang, 2014). They both showed antitumor activities and were used to treat acute myeloid leukemia (AML) and chronic myeloid leukemia (CML; Takeda et al., 1982; Boyd and Sullivan, 1984; Zhang et al., 2016). HT could block peptide bond formation and aminoacyl-tRNA binding to inhibit protein synthesis (Fresno et al., 1977). In addition to antileukemic effects, HT was also reported to exhibit antiviral activity in recent years. HT inhibited chikungunya virus (CHIKV) replication by suppressing viral protein synthesis (Kaur et al., 2013). Antiviral activity of HT against Singapore grouper iridovirus (SGIV), varicella-zoster virus (VZV), and Zika virus (ZIKV) was demonstrated as well (Jia et al., 2018; Kim and Song, 2019; Lai et al., 2020). However, whether HT has an anti-HSV-1 effect is still unknown. In our current study, we investigated if HT inhibits HSV-1 replication and the potential mechanisms.

## Materials and Methods

### Cells and Viruses

Vero cells were cultured and maintained in Dulbecco’s modified Eagle’s medium (DMEM, Gibco) supplemented with 10% fetal bovine serum (FND500, ExCell Bio, Shanghai, China) in a humidified 37°C 5% CO_2_ incubator. HSV-1 HF and HSV-1 GFP, initially obtained from Professor Erguang Li of Nanjing University, were propagated in Vero cells. HSV-1 McKrae and HSV-1 F were kindly provided by Dr. Kai Hu, Department of Ophthalmology, Nanjing Drum Tower Hospital. HSV-1 blue, a thymidine kinase (TK) mutant derived from HSV-1 KOS, was a gift from Dr. Tao Peng of State Key Laboratory of Respiratory Disease, Guangzhou Institutes of Biomedicine and Health, Chinese Academy of Sciences, and an ACV-resistant clinical HSV-1 strain HSV-1 153 was a kind gift from Prof. Yifei Wang, Institute of Biomedicine, College of Life Science and Technology, Jinan University.

### Antibodies and Reagents

Harringtonine was purchased from MCE (Cat#: HY-N0862, NJ, United States) and dissolved in Dimethyl Sulphoxide at a concentration of 10mM. ACV was purchased from Selleck (Cat#: S1807, Houston, TX, United States). Antibodies against gD (Cat#: sc-69802), ICP4 (Cat#: sc-69809), and HVEM (Cat#: sc-365971) were purchased from Santa Cruz Biotechnology (Santa Cruz, CA, United States), and anti-GAPDH antibody was purchased from Cell Signaling Technology (CST, MA, United States).

### Antiviral Activity

The anti-HSV-1 activity of HT or ACV was determined by In-cell Western assay. Vero cells grown in 96-well plates were treated with indicated concentrations of the test compounds for 30min at 37°C. The cells were then infected with HSV-1 multiplicity of infection (MOI=1) with the presence of a test compound. After further incubation for 24h, the supernatant was removed, and the cells were measured by In-cell Western assay of HSV-1 gD protein expression. The 50% inhibitory concentration (IC_50_) for antiviral activity was calculated as the concentration of antiviral compounds that reduce the virus-induced infection by 50% comparing to the virus infection without compound treatment.

### In-Cell Western Assay

According to the manufacturer’s instructions, the In-cell Western assay was carried out using the Odyssey Imaging System (Li-COR Biosciences, NE, United States). Vero cells cultured in a 96-well plate were either mock-infected or infected with HSV-1 at a given MOI when they reached 80% cell density. After treatment, they were fixed with 4% paraformaldehyde for at least 30min. Then, the cells were permeabilized with 0.5% Triton X-100 for 15min and blocked with blocking buffer (4% non-fat dry milk) for 90min and incubated with gD antibody diluted in blocking buffer (1:500) at 4°C overnight. After being washed three times with PBST, the cells were stained with IRDye IgG (1:2,000) for 1h following 1h DRAQ5 staining and scanned with an Odyssey Infrared Imager. The relative amount of gD protein expression was obtained by normalizing to endogenous DRAQ5 in all experiments. Viral inhibition (%) was calculated as follows: [1−fluorescence_gD_/fluorescence_control_] * 100%, where “fluorescence_gD_” indicates gD protein expression of the infected cells with the compound treatment and “fluorescence_control_” indicates gD protein expression of the infected cells without the compound treatment.

### Time-of-Drug Addition Assay

Vero cells grown in the 96-well plate were inoculated in triplicate with HSV-1 HF (MOI=1). HT at 0.5μM was treated at −2 (2h before viral inoculation), 0 (instantaneous viral inoculation), 2, 4, 6, 8, 12, and 24h followed by infection with HSV-1. Viral protein level was determined in 96-well plate by In-cell Western assay at the indicated time points.

### Virucidal Assay

Herpes simplex virus type 1 HF (1*10^7^ PFU) was diluted in 1ml DMEM at 37°C with or without HT at the indicated concentrations for 2h. After the treatment, the mixture was diluted to 1,000-fold to obtain an equivalent of 10^4^ PFU in 1ml DMEM. HT also was diluted to a non-inhibitory concentration against HSV-1 at this step. The diluted mixture was added to confluent Vero cell monolayers in a 96-well plate and incubated at 37°C for 24h. At the same time, a group of HT at the indicated concentrations was incubated at 37°C for 2h, which was then directly added to the confluent Vero cell monolayer without dilution. After incubation for 24h, the supernatant was removed, and the infected cells were measured by In-cell Western assay.

### Determination of 50% Tissue Culture Infectious Dose

Vero cells were infected with HSV-1 (MOI=1) with or without HT. About 2h post-infection (hpi), the supernatant was removed, and the new DMEM containing 2% FBS was added, and the culture was maintained for 24h. Then, 10-fold serially dilutions of the virus suspension were used to infect monolayer Vero cells in a 96-well plate for 72h. Each dilution was tested with eight replicates in each experiment, and the wells were observed and scored for the presence or absence of cytopathic effect (CPE). The 50% tissue culture infectious dose (TCID_50_) was calculated based on the Reed–Muench method (Capelli et al., 2020).

### Western Blot Assay

The cells were lysed by adding RIPA lysis buffer supplemented with protease inhibitors in an ice bath for 10min. The lysates were centrifuged at 12,000×*g* for 10min at 4°C, and the supernatants containing total proteins were prepared. Total protein concentrations were determined by the BCA protein assay kit (Pierce, Rockford, IL, United States). The proteins were separated by SDS-PAGE and transferred onto polyvinylidene fluoride (PVDF) membranes. Proteins were detected by the respective primary antibodies overnight at 4°C and then IRDye IgG (1:10,000 dilution) for 1h. Bands were visualized under Li-COR Odyssey Infrared Imager (Li-COR).

### RNA Extraction and qPCR Analysis

Total RNA was extracted using TRIzol reagent (Life Technologies), and equivalent amounts of RNA (1μg) from each sample were subjected to reverse-transcription using a PrimeScript®RT reagent kit (Takara, Japan) according to the manufacturer’s protocol. Quantitative real time-PCR (qPCR) was performed using the ABI SYBR Green Master Mix (Life Technologies), followed by detecting an ABI Prism 7500 Sequence Detection System. GAPDH was used for the normalization of mRNA, and analysis was carried out using the 2^−ΔΔ*C*t^ method. The sequences of primers used in this study are listed in Table 1.

**Table 1:** Primers were used in this study.

Targets	Forward (5'–3')	Reverse (5'–3')
HSV-1 gD	AGC AGG GGT TAG GGA GTT G	CCA TCT TGA GAG AGG CAT C
HSV-1 ICP4	GGC CTG CTT CCG GAT CTC	GGT GAT GAA GGA GCT GCT GTT
HVEM	CTG CTC CAG GAC AGA GAA C	CGG AGA CGA TCA CCT TGA C
Nectin-1	CTG CAA AGC TGA TGC TAA CC	GAT GGG TCC CTT GAA GAA GA
Nectin-2	GAG GAC GAG GGC AAC TAC AC	AGG GAT GAG AGC CAG GAG AT
3-OST-3	CAG GCC ATC ATC ATC GG	CCG GTC ATC TGG TAG AA
GAPDH	GAG TCA ACG GAT TTG GTC GT	CTT GAT TTT GGA GGG ATC TCG C

### Cytotoxicity Assay

Vero cells were seeded in 96-well plates and incubated at 37°C, 5% CO_2_ overnight. The supernatant was then removed, and a new medium with increasing doses of HT or ACV was added. After treatment for 48h, the medium was replaced with 100μl of fresh medium containing 10μl of Cell Counting Kit 8 (CCK-8) reagent (Dojindo Laboratories, Kumamoto, Japan) for 2h. OD values at 450nm were measured by a TECAN Infinite M200 microplate reader (Männedorf, Switzerland). The cell viability values for treated cells were normalized with those of untreated cells. The 50% cytotoxic concentration (CC_50_) was calculated as the concentration of compounds that reduce 50% cell viability comparing to the cells without compound treatment.

### Immunofluorescence Assay

Vero cells were seeded on coverslips in 12-well plates. Cells were uninfected or infected with HSV-1 GFP (MOI=1) in the presence of HT. After 24h, cells were fixed with 4% paraformaldehyde for 30min at RT, permeabilized with 0.1% Triton X-100 for 15min, and blocked with 2% BSA for 1h. Cellular proteins were immunolabeled with anti-HVEM antibody overnight at 4°C and then Alexa Fluor 594 (red)-labeled secondary antibody (Life Technologies) for 1h. Nuclei were stained with DAPI (blue). Finally, images were obtained using an Olympus FluoView FV10i confocal microscope (Tokyo, Japan).

### Statistical Analysis

Statistical analysis was performed with one-way ANOVA or Student’s *t*-test as appropriate, using GraphPad Prism 5 (Version 5.01, GraphPad Software). The level of significance was set at ^*^p<0.05, ^***^p<0.01, or ^***^p<0.001.

## Results

### Cytotoxicity and Anti-HSV-1 Activity of HT

Vero cells were treated with increasing concentrations of HT and then infected with five different HSV-1 strains, including two ACV-resistant isolates, to determine the effect of HT on HSV-1 infection. ACV was chosen as a positive control in the antiviral experiment. After 24h, the antiviral effects of HT and ACV were determined by In-cell Western assay, and the IC_50_ values were calculated. As shown in Table 2, HT IC_50_ values range from 0.1081 to 0.1584μM. HT showed higher antiviral activity for all five HSV-1 strains at the same concentration of ACV and HT than ACV. These results suggested that HT could efficiently inhibit HSV-1 infection. The cytotoxicity of HT and ACV in Vero cells was detected by CCK8 assay (Figure 1B), and the CC_50_ value of HT in Vero cells was 239.6μM, as shown in Table 2. The selective index (SI) of HT against HSV-1 HF, HSV-1 F, HSV-1 Mckrae, HSV-1 blue, and HSV-1 153 was 2216.5, 1871.9, 1973.6, 1815.2, and 1512.6 in Vero cells, respectively.

**Table 2:** Harringtonine was effective against HSV-1 infection including five HSV-1 strains.

	Virus	IC_50_ (μM)	CC_50_ (μM)	SI
HT	HSV-1 HF	0.1081±0.013	239.6±26.3	2,216.47
	HSV-1 F	0.1280±0.011		1,871.88
	HSV-1 Mckrae	0.1214±0.014		1,973.64
	HSV-1 blue	0.1320±0.007		1,815.15
	HSV-1 153	0.1584±0.009		1,512.63
ACV	HSV-1 HF	1.2570±0.081	>1,000	>795.54
	HSV-1 F	0.2085±0.016		>4,796.16
	HSV-1 Mckrae	0.5183±0.019		>1,929.38
	HSV-1 blue	>50		Uncertain
	HSV-1 153	>50		Uncertain

IC_50_, the half maximal inhibitory concentration; CC_50_, the half maximal cytotoxicity concentration; SI, selectivity index; and CC_50_/IC_50_. Values represent the mean±SD of three independent experiments.

### HT Showed Effective Antiviral Activity Against HSV-1

**Figure 2:**
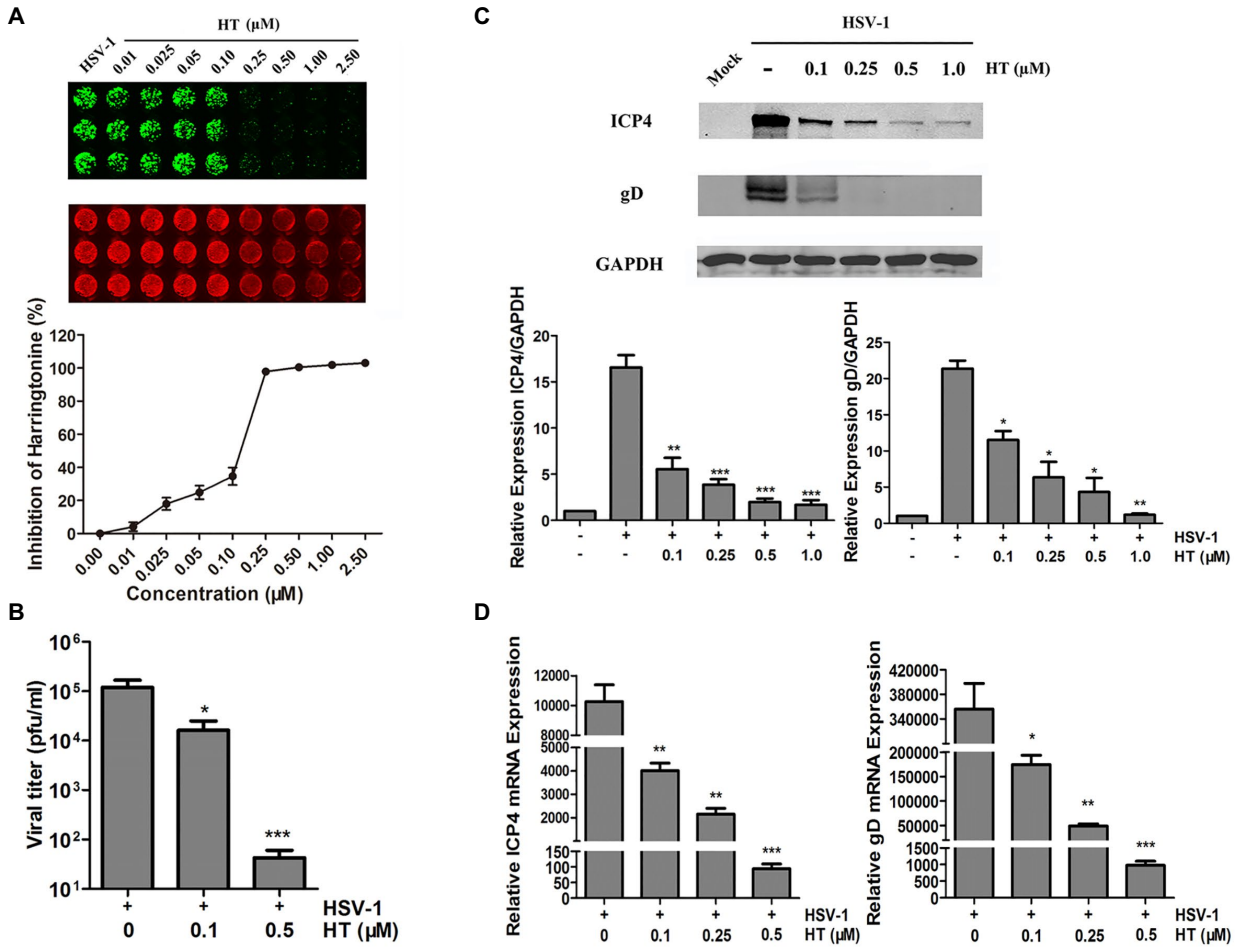
Anti-herpes simplex virus type 1 (HSV-1) activity of HT. **(A)** Vero cells were inoculated with HSV-1 HF and treated with different concentrations of HT for 24h. Viral protein glycoprotein D (gD; green) was determined by In-cell western and normalized by DRAQ5. DRAQ5 (red) is a far-red DNA stain for fluorescent cellular imaging applications with live cells. **(B)** Vero cells seeded in six-well plates were infected with HSV-1 HF (MOI=1) in the presence of HT at different concentrations (0.1 and 0.5μM) for 24h. Viral titers in the medium were determined by half maximal tissue culture infective dose (TCID_50_). **(C)** Vero cells in six-well plates were mock-infected or infected with HSV-1 HF (MOI=1) after HT was incubated at the indicated concentrations (0.1, 0.25, 0.5 and 1.0μM). Cells were collected after 24h. Total protein levels were quantified *via* Western Blot assay, where the samples were immunoblotted for ICP4 and gD protein in non-infected, infected and infected as well as treated with different concentrations of HT. **(D)** HSV-1 ICP4 and gD transcripts were determined by quantitative real time-PCR (qPCR). HSV-1 infection was performed as mentioned in **(C)**. Cells were collected at 24h to extract RNA, and viral transcripts were quantified by qPCR and represented as fold change to the uninfected cells. Student’s *t*-test was used to compare transcripts levels of viral proteins as well as viral titer assay in non-treated and treated cells. ^*^p<0.05, ^**^p<0.01, and ^***^p<0.005.

Next, we further evaluated the effect of HT on the HSV-1 HF strain. Vero cells were infected with HSV-1 at 1 MOI in the presence of increasing concentrations of HT for 24h. Then, gD protein was measured by In-cell Western assay. As shown in Figure 2A, HT significantly inhibited HSV-1 infection at 0.25–2.5μM concentrations. A progeny virus yield assay was performed to evaluate the effect of HT on HSV-1 virus proliferation. Vero cells were infected with HSV-1 at 1 MOI in the presence of 0, 0.1, and 0.5μM HT for 2h, and the inoculum was replaced with a medium containing HT at the corresponding concentrations for another 24h. The supernatant was collected and added to Vero cell monolayers at 10-fold serially dilutions. Then, we determined the virus titers by a TCID_50_ assay. As shown in Figure 2B, in untreated-HSV-1-infected cells, the virus titers of HSV-1 were 5.08±0.23 log_10_ PFU/ml, while in the presence of HT at 0.1 and 0.5μM, the virus titers of HSV-1 were 4.21±0.42 log_10_ PFU/ml and 1.63±0.29 log_10_ PFU/ml, respectively. HT at 0.1μM resulted in 0.87 log reduction of HSV-1 titers, and HT at 0.5μM resulted in 3.45 log reduction of HSV-1 titers. HT significantly reduced HSV-1 titers in a dose-dependent manner.

Viral gene and protein expression were also determined to examine the inhibitory activity of HT. Vero cells were infected with HSV-1 HF in the presence of HT at increasing concentrations. After 24h, cells were collected, and cellular proteins were extracted for Western Blot and RNA for qPCR. HSV-1 immediate-early protein ICP4 (infected cell protein 4) and HSV-1 late protein gD (glycoprotein D) were detected in the HSV-1-infected-Vero cells. The results showed that HT reduced the protein levels of viral ICP4 and gD in a dose-dependent manner (Figure 2C). Similar results of ICP4 mRNA and gD mRNA were also obtained in Figure 2D, correlated with Figure 2C.

### HT Showed a Stronger Anti-HSV-1 Effect Than ACV

**Figure 3:**
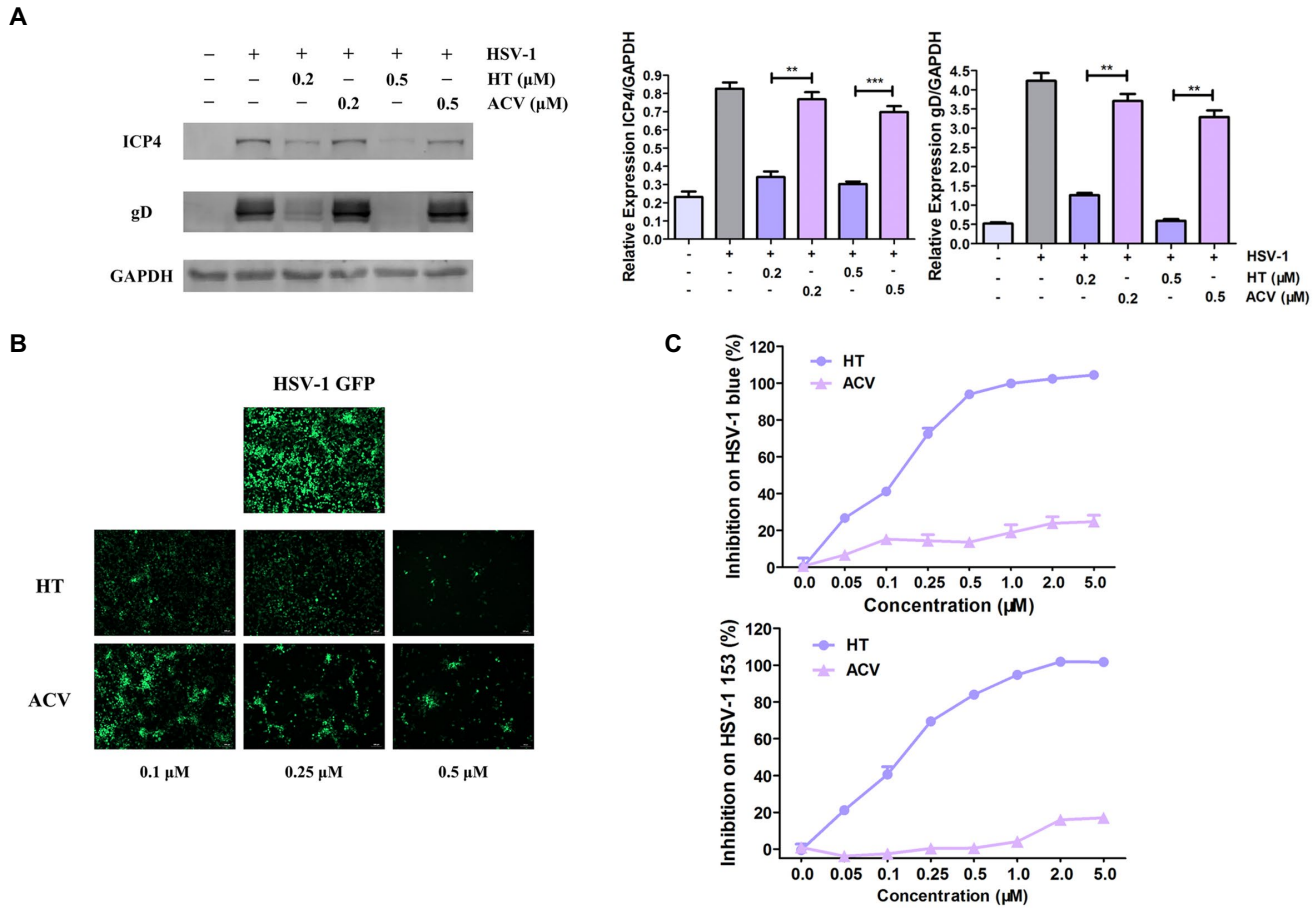
Harringtonine had a better antiviral effect than ACV. **(A)** Vero cells were treated with either HT or ACV at two different concentrations including 0.2 and 0.5μM and then infected with HSV-1 HF (MOI=1). The cells were harvested at 24h, then viral protein ICP4 and gD were determined by Western Blot assay. **(B)** Vero cells were infected with HSV-1 GFP (MOI=1) and treated with either HT or ACV at the indicated concentrations for 24h, respectively. Images were obtained in the presence of HT or ACV concentration ranged from 0.25 to 0.50μM in HSV-1 GFP-infected-Vero cells. The inhibitory effects on viral fluorescent protein expression were observed under a fluorescence microscope. **(C)** HT also displayed antiviral efficacy on ACV-resistant HSV-1 strains including HSV-1 blue and HSV-1 153. Vero cells were infected with HSV-1 blue and HSV-1 153 (MOI=1) in the presence of HT or ACV (0.05, 0.1, 0.25, 0.5, 1.0, 2, and 5μM) for 24h, respectively. The antiviral effects were determined by In-cell Western assay. The data was presented as means±SD. One-way ANOVA was used to compare viral protein ICP4 and gD in Vero cells at different concentrations of HT-treated cells with ACV-treated cells. ^**^p<0.01 and ^***^p<0.005.

The most potent antiviral drug currently used to treat HSV-1 is ACV, a nucleoside analog (De Clercq and Field, 2006). Firstly, to compare the antiviral effects of HT with ACV, Vero cells were treated with the same concentrations of HT and ACV, and then they were infected with HSV-1 HF (MOI=1) for 24h. Western Blot assay revealed that viral proteins ICP4 and gD were decreased by more than 50 and 80% in HT-treated-HSV-1-infected cells, respectively. However, the proteins of ICP4 and gD were reduced by only 20% in ACV-treated-HSV-1-infected cells (Figure 3A). Vero cells were also infected with HSV-1 GFP at 1 MOI in the presence of HT and ACV, respectively. After 24h, HT-treated cells and ACV-treated cells were imaged by a fluorescence microscope. We found that the fluorescence intensity of HT-treated cells was markedly weaker than that of ACV at all corresponding concentrations, which indicated that HT inhibited HSV-1 replication more potently than ACV (Figure 3B).

We further evaluated the antiviral effect of HT against ACV-resistant strains, including HSV-1 blue and HSV-1 153 (Wang et al., 2011). In the presence of HT or ACV, Vero cells were infected with HSV-1 blue or HSV-1 153 for 24h, and the viral protein gD was analyzed by In-cell Western assay, and the bands’ density was scanned by a densitometer. As shown in Figure 3C, the IC_50_ and IC_90_ values of HT inhibition of HSV-1 blue infection were 0.1320 and 0.7832μM, respectively, and the IC_50_ and IC_90_ values for HSV-1 153 were 0.1584 and 1.0134μM, respectively. As expected, both viruses resisted ACV inhibition at a concentration up to 5μM, consistent with previous reports (Li et al., 2019). These results demonstrated that HT not only more potently inhibited HSV-1 replication but also suppressed ACV-resistant strains HSV-1 blue and HSV-1 153.

### HT Inhibited HSV-1 at an Early Stage

**Figure 4:**
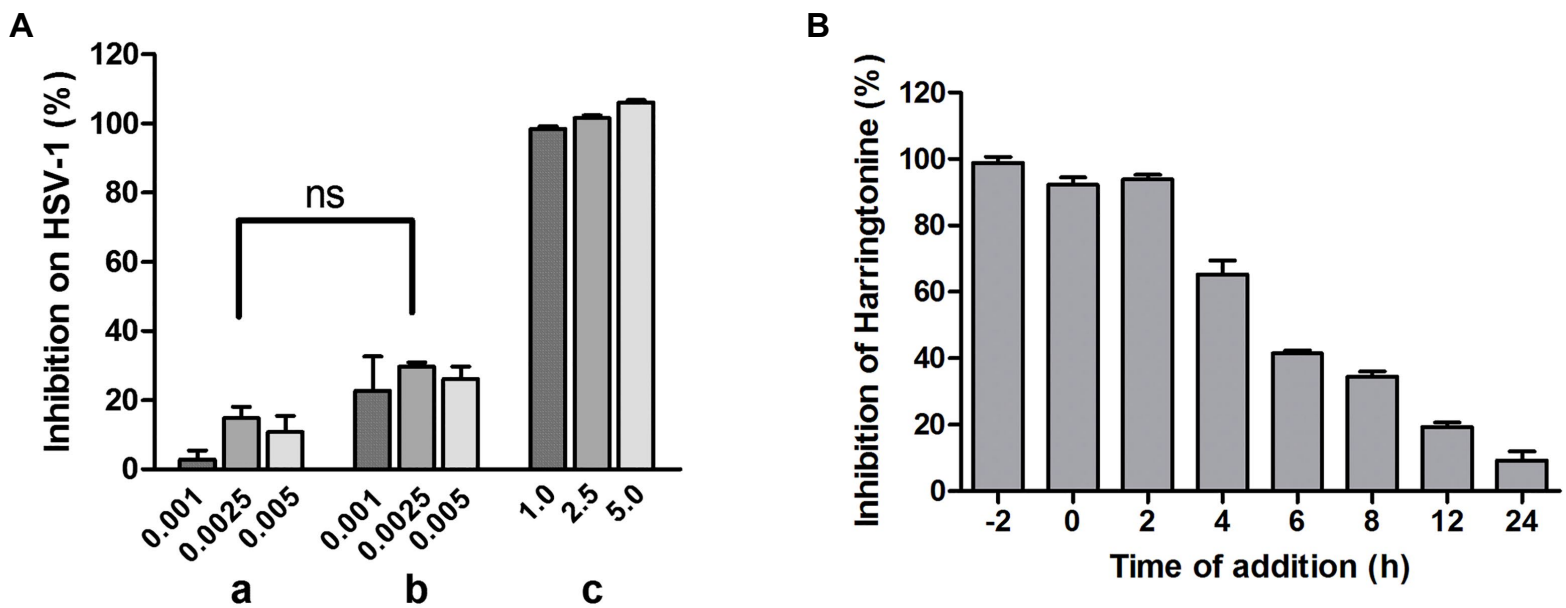
**(A)** No virucidal activity of HT against HSV-1. In group **(a)**, HSV-1 was incubated with 1.0, 2.5, and 5.0μM HT at 37°C for 2h. After HT treatment, the mixture was diluted to a un-inhibitory concentration of HT (0.001, 0.0025, and 0.005μM), about 1,000-fold dilution, and plated on Vero cells for 24h. In group **(b)** and **(c)**, HSV-1 was incubated in the absence of HT at 37°C for 2h. HSV-1 was diluted to 1,000-fold and plated on Vero cells in the presence of HT at 1.0, 2.5, and 5.0μM **(c)** or 1,000-fold dilution of HT at 1.0, 2.5, and 5.0μM **(b)** for 24h. The viral protein gD expression was determined by In-cell western assay. Group **(a)** and Group **(b)** showed no significant difference. **(B)** Time-of-addition assay. Vero cells were treated with HT at 0.5μM at different time points after infection with HSV-1 HF [−2, 0, 2, 4, 6, 8, 12, and 24 post-infection (hpi)]. At 24hpi, the antiviral effect of HT was detected using In-cell Western assay. Data are presented as mean±SD of three independent experiments.

To investigate whether HT directly inactivates HSV-1 virions, we performed a virucidal assay. In group a, HSV-1 was co-incubated with HT at inhibitory concentrations of 1.0, 2.5, and 5.0μM, respectively, at 37°C for 2h, and the mixtures were diluted to non-inhibitory concentrations of 0.001, 0.0025, and 0.005μM of HT and were added to Vero cells. In group b, HSV-1 was incubated alone at 37°C for 2h before being diluted to the same viral inoculum as group a, and then the virions were added to Vero cells with HT at 0.001, 0.0025, 0.005μM. In group c, HSV-1 infection was performed as group b, but the concentration of HT was adjusted to viral inhibitory concentrations of 1, 2.5, and 5μM, respectively. In-cell Western assay was performed to detect the viral gD protein expression after 24h. There was no difference between groups a and b, which showed that the infectivity of HT-virus co-incubation was similar to that of HT and virus without co-incubation (Figure 4A). This result suggested that the pre-treatment of the virus with HT had no virucidal effect on HSV-1 virions. Next, to explore the potential mechanism of HT antiviral activity, we conducted the time-of-addition assay. HT at 0.5μM was added to Vero cells at various time points of −2, 0, 2, 4, 6, 8, 12, and 24hpi. After treatment for another 2h, viral protein gD was detected by In-cell Western assay. As shown in Figure 4B, HSV-1 replication was almost completely inhibited when HT was added at −2, 0, and 2hpi. However, with the time lag of the drug, the antiviral effect gradually decreased. This result suggested that HT may act at an early stage of HSV-1 replication.

### HT Reduced Expression of HVEM

**Figure 5:**
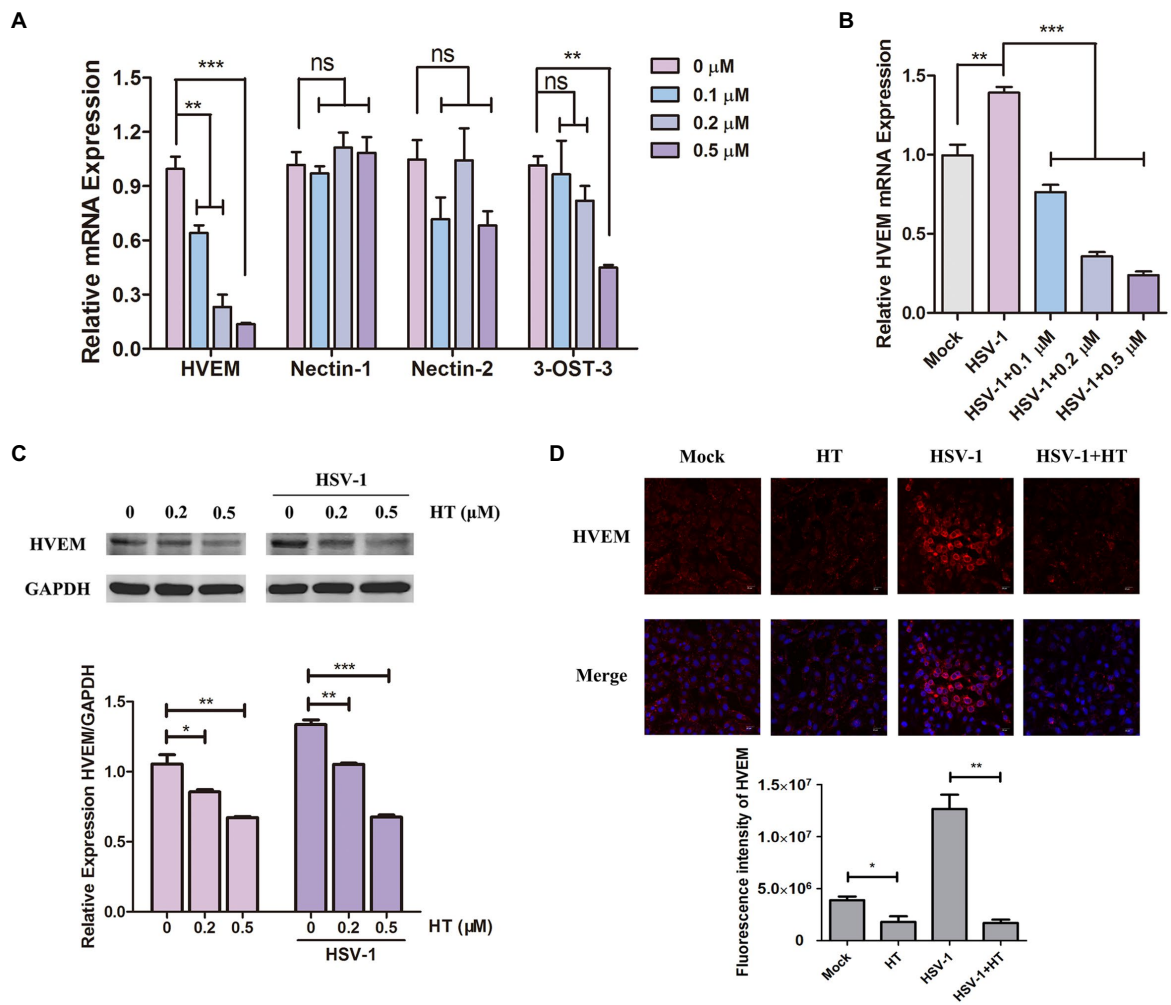
Harringtonine reduced herpes virus entry mediator (HVEM) expression. **(A)** Vero cells were mock-treated or treated with HT at different concentrations including 0.1, 0.2, and 0.5μM for 4h. The mRNA levels of HVEM, nectin-1, nectin-2, and 3-OST-3 were determined by qPCR analysis and represented as fold change to mock-treated cells. **(B)** Vero cells were infected with HSV-1 (MOI=1) in the presence of the HT at different concentrations including 0.1, 0.2, and 0.5μM for 4h. The mRNA level of HVEM was determined by qPCR analysis and represented as fold change to the uninfected cells. **(C)** Vero cells in six-well plates were treated with in the absence or in the presence of HT at 0.2 and 0.5μM for 4h and HT-treated Vero cells at the same time were infected with HSV-1. The HVEM protein was determined after 4h by Western Blot assay. **(D)** Vero cells were incubated in the absence or in the presence of HT treatment at 0.5μM for 4h. The HT-treated Vero cells were infected with HSV-1 HF (MOI=1) for 4h at the same time. The Vero cells were then stained for HVEM (red) and DAPI (nucleus, blue), respectively. Representative confocal microscopy images were obtained. Scale bars=10μm. ^*^p < 0.05, ^**^p < 0.01, and ^***^p < 0.005.

The early steps of HSV-1 infection include adsorption, entry, and intracellular transport. Membrane fusion starting from gD binding to the specific cellular receptors is a critical step of HSV-1 entry into host cells (Nicola et al., 2005). We investigated the effect of HT on four major gD receptors, including HVEM, nectin-1, nectin-2, and 3-O-S HS. 3-OST-3 is the particular enzyme that generates the modified form of 3-OS HS (Shukla et al., 1999). Vero cells were treated with HT at the indicated concentrations for 4h, and the mRNA levels of HVEM, nectin-1, nectin-2, and 3-OST-3 were measured by qPCR analysis. Results showed that HVEM mRNA levels were significantly reduced after treatment with different concentrations of HT. However, the mRNA levels of nectin-1 and nectin-2 were not affected significantly in the presence of HT. At the highest concentration of 0.5μM but not at the lower concentrations of 0.1 and 0.2μM, the mRNA level of 3-OST-3 was reduced by the treatment of HT. Only HVEM was downregulated in HT-treated cells in a dose-dependent manner, and it showed a decrease of HVEM about 40% at 0.1μM, 70% at 0.2μM, and 85% at 0.5μM compared to untreated cells (Figure 5A). After HSV-1 infection, the mRNA level of HVEM was upregulated, as shown in Figure 5B. While in the presence of HT, HVEM was decreased in HSV-1-infected-cells, and HT also showed a dose-dependent suppression of HVEM (Figure 5B). We further examined the protein levels of HVEM after HT treatment by Western Blot analysis. HT at 0.2μM reduced 20% of HVEM protein, and HT at 0.5μM reduced 40% of HVEM compared to the untreated cells. Similar results of WB showed that HVEM was upregulated with the infection of HSV-1. After treatment with HT, HVEM was suppressed in HSV-1-infected-cells compared to untreated cells (Figure 5C).

Moreover, HT-induced downregulation of HVEM protein was further confirmed by immunofluorescence assay. As shown in Figure 5D, the fluorescence intensity of HVEM was reduced in either HSV-1-infected or uninfected cells after HT treatment. These results together suggested that inhibition of HVEM by HT may interrupt viral membrane fusion and virus entry. Taken together, HT blocked the entry receptor HVEM of HSV-1, thereby affecting the viral replication.

## Discussion

Due to the high prevalence of HSV-1, the drug-resistant mutations, and the lack of vaccines, there is an urgent need to develop novel antiviral agents against HSV-1. Our study demonstrated that HT had antiviral activities against five different HSV-1 strains, including two ACV-resistant strains. HT exhibited an effective inhibition of various isolates of HSV-1, with IC_50_s in the range of 0.1–0.5μM, slightly better than ACV (Table 2). ACV has been widely used for the majority of HSV-1-related infections. ACV is phosphorylated by the viral TK and then becomes incorporated into the replicating viral DNA strand and hinders the function of the viral DNA polymerase (Arduino and Porter, 2006). More recently, an increasing number of inhibitors of viral DNA synthesis and mostly derivatives of ACV, such as ganciclovir and valaciclovir, have been used for clinical treatment. However, due to the prolonged use of these drugs, the limited availability of drug options, and ACV application in immunocompromised patients, drug-resistant mutations are increasingly common. A study reported a proportion of 6.4% ACV-resistant HSV-1 isolated from immunocompetent patients with herpes keratitis (Duan et al., 2008). Among immunocompromised patients, the proportion of ACV resistance is higher and ranges from 3.5 to 10% (Piret and Boivin, 2011). There is a need to develop new compounds with different antiviral actions against ACV-resistant HSV-1. Our results showed that HT had significant inhibitory effects on HSV-1 resistant strains, including HSV-1 blue and HSV-1 153, both of which are TK mutations (Figure 3C). Considering that the potential antiviral mechanism of HT differs from ACV, HT is a promising candidate for further development as a therapeutic agent for ACV-resistant HSV-1 infection.

Mechanistically, HT reduced HVEM expression leading to the significant downregulation of a key entry receptor for HSV-1. A recent study reported that HHT could exhibit antiviral activity against HSV-1 by antagonizing the phosphorylation level of eukaryotic initiation factor 4E (p-eIF4E; Dong et al., 2018). However, in our study, HT did not affect eIF4E phosphorylation (Supplementary Figure S1), suggesting that HT has a different antiviral action from HHT. HT did not exhibit virucidal activity against HSV-1, which means HT has no direct killing effect on the virion of HSV-1 (Figure 4A). It is thus inferred that the antiviral activity of HT may occur on host factors. The time-of-drug assay was conducted to explore the mechanism of action of HT, and we found that HT mainly affected the early stages of viral infection (Figure 4B). Initial infection of HSV-1 starts with binding to and then entry into host cells. The entry of HSV-1 is critical for subsequent infection and occurs by interactions between viral components and cell surface receptors (Spear, 2004). There are four viral glycoproteins gB, gD, gH, and gL, required for HSV-1 to enter into host cells, and binding a cellular receptor specific to gD is necessary (Pertel et al., 2001; Heldwein and Krummenacher, 2008). The most widely used gD receptors are HVEM and nectin-1, as HVEM is found to be used by all the tested clinical isolates of HSV-1, as well as nectin-1 (Krummenacher et al., 2004). Once HSV-1 starts to exploit the receptors for entry, an infection is ready to be established, followed by penetrating the cell (Banerjee et al., 2020). Therefore, inhibition of HVEM prevents HSV-1 from entering the host cells and reduces infection of HSV-1. In our study, nectin-1 and nectin-2 were not downregulated by HT, while 3-OST-3 was only reduced at a high concentration of HT at 0.5μM. However, HVEM expression was significantly suppressed by HT at concentrations that resulted in significant viral inhibition but did not affect 3-OST-3 expression (Figure 5A), suggesting that HVEM plays a key role in HSV-1 entry in our *in vitro* cell assays. Interestingly, at both the protein level and mRNA level, HSV-1 infection upregulated the HVEM expression, which was reversed by HT treatment (Figures 5B–D), indicating a potential positive regulation loop for the HSV-1 infection. According to these data, we postulate that HT prevents HSV-1 entry by targeting the gD receptor HVEM.

As an immune factor, HVEM also plays a role in the pathology of HSV-1 infection. During the chronic inflammation of herpes simplex keratitis (HSK), expressions of HVEM and its ligands (LIGHT, BTLA, and CD160) on T cells promote infiltration of inflammatory cells in the cornea and corneal sensitivity loss (Edwards et al., 2017; Park et al., 2020). It has been shown that HVEM was upregulated during latency and the immune function of HVEM contributed to the establishment of HSV-1 latency and reactivation, which was independent of gD binding (Wang et al., 2018; Tormanen et al., 2020). Thus, inhibition of HVEM by HT would prevent HSV-1 latency and reactivation, which is one of the most challenging issues for clinical treatment.

In conclusion, our study demonstrated that HT had potent inhibitory activity against HSV-1 infection against both WT and ACV resistant isolates through suppression of HVEM. The low cytotoxicity and broadly potent anti-HSV-1 activity make HT a promising therapeutic candidate for HSV-1 treatment.

## Data Availability Statement

The original contributions presented in the study are included in the article/Supplementary Material, further inquiries can be directed to the corresponding authors.

## Author Contributions

DC and YL designed the project and prepared the manuscript. YL performed most of the experiments *in vitro*. QY and FZ helped to prepare HSV-1. DC, YL, ZH, and ZW analyzed the data. ZW supervised and financially supported the study and revised the manuscript. All authors contributed to the article and approved the submitted version.

## Conflict of Interest

The authors declare that the research was conducted in the absence of any commercial or financial relationships that could be construed as a potential conflict of interest.

## Publisher’s Note

All claims expressed in this article are solely those of the authors and do not necessarily represent those of their affiliated organizations, or those of the publisher, the editors and the reviewers. Any product that may be evaluated in this article, or claim that may be made by its manufacturer, is not guaranteed or endorsed by the publisher.
