# Supplementary figures and images for: Harringtonine Inhibits Herpes Simplex Virus Type 1 Infection by Reducing Herpes Virus Entry Mediator Expression
Source: Front Microbiol. 2021 Aug 31;12:722748. doi: 10.3389/fmicb.2021.722748 (PMC8438530; doi:10.3389/fmicb.2021.722748)

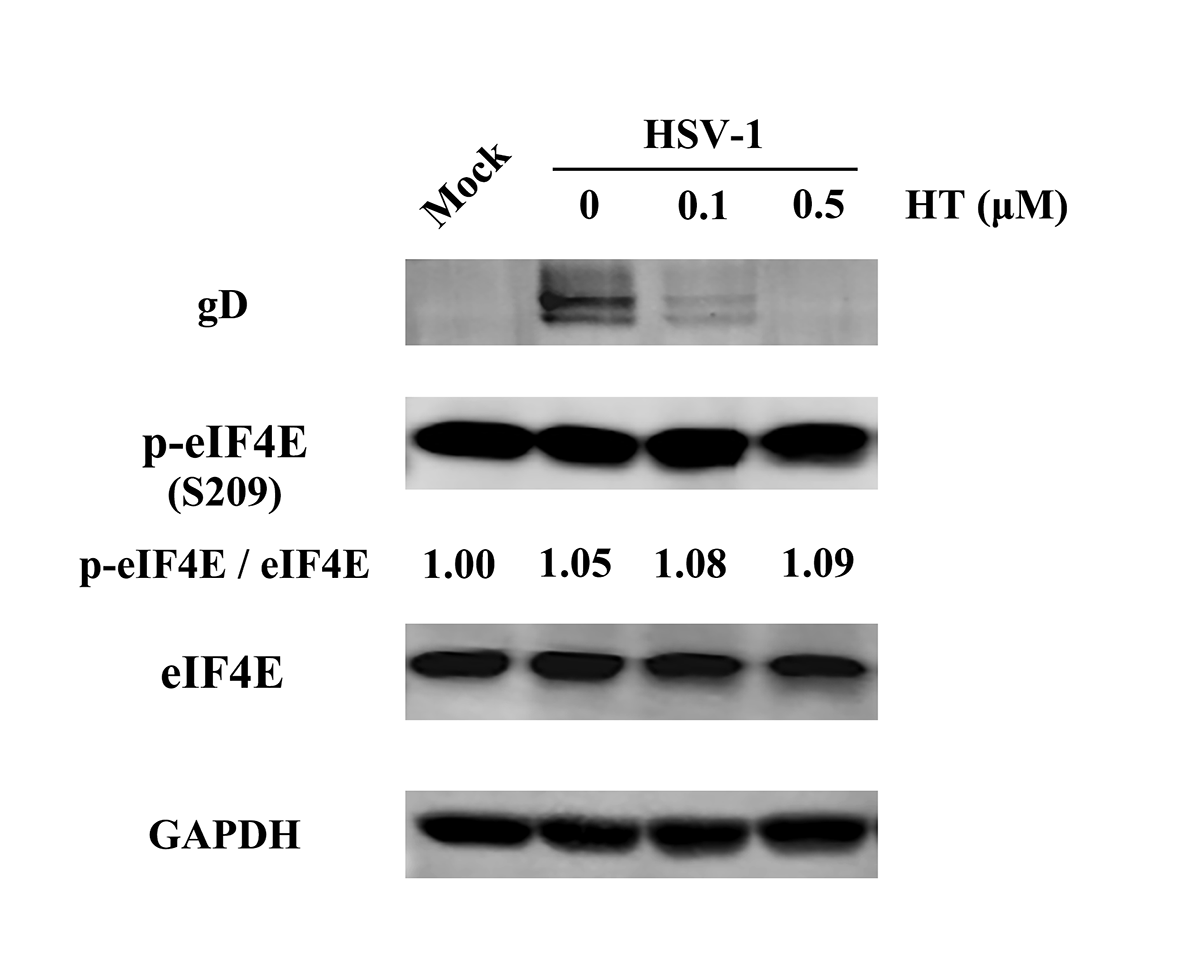

Supplement: Supplementary file 1 [file Image_1.TIF]
