# Supplementary material for: Harringtonine Inhibits Herpes Simplex Virus Type 1 Infection by Reducing Herpes Virus Entry Mediator Expression
Source: Front Microbiol. 2021 Aug 31;12:722748. doi: 10.3389/fmicb.2021.722748 (PMC8438530; doi:10.3389/fmicb.2021.722748)
Supplement: Supplementary file 2 [file Data_Sheet_1.docx]

**Figure S1**. HT did not inhibit the phosphorylation of eIF4E (p-eIF4E). Vero cells were infected with HSV-1 HF (MOI = 1) in the presence of HT or HHT at indicated concentrations, respectively. After 24 h p.i, the proteins p-eIF4E (at Ser 209) and eIF4E were detected by Western Blot assay.
